# Outcome of iron reduction therapy in ex-thalassemics

**DOI:** 10.1371/journal.pone.0238793

**Published:** 2021-01-22

**Authors:** Fouzia N. Aboobacker, Gaurav Dixit, Kavitha M. Lakshmi, Anu Korula, Aby Abraham, Biju George, Vikram Mathews, Alok Srivastava

**Affiliations:** Department of Haematology, Christian Medical College, Vellore, India; Nihon University School of Medicine, JAPAN

## Abstract

There is limited data on iron reduction therapy (IRT) after successful allogeneic haematopoietic stem cell transplantation (aHSCT) for patients with thalassemia major (TM). We present the long term outcome of IRT in 149 patients with TM who underwent aHSCT during January, 2001-December, 2012. The median age was 7 years (range:1–18) and 92 (61.7%) belonged to Pesaro class 3 with a median ferritin at aHSCT of 2480ng/ml (range:866–8921). IRT was reinitiated post-aHSCT at a median of 14 months (range:5–53) post aHSCT with phlebotomy alone in 10 (6.7%) patients or iron chelation alone in 60 (40.3%) patients while 79 (53%) were treated with the combination. Reduction in serum ferritin/month [absolute quantity (ng/ml/month) was as follows: 87 (range:33–195), 130 (range:17–1012) and 147 (range:27.7–1427) in the phlebotomy, chelation and combination therapy groups, respectively (p = 0.038). With a median follow up of 80 months (range:37–182), target ferritin level of <300ng/ml was achieved in 59(40%) while a level <500ng/ml was achieved in 88 patients (59%) in a median duration of 41 months of IRT (range: 3–136). Patients in class III risk category and higher starting serum ferritin levels (>2500ng/ml) were associated with delayed responses to IRT. Our data shows that IRT may be needed for very long periods in ex-thalassaemics to achieve target ferritin levels and should therefore be carefully planned and initiated as soon as possible after aHSCT. A combination of phlebotomy and iron chelators is more effective in reducing iron overload.

## Introduction

All patients with thalassemia major (TM) receive regular blood transfusion with or without chelation before undergoing allogeneic haematopoietic stem cell transplantation (aHSCT). aHSCT now cures >80% of thalassemia patients. ‘Ex-thalassaemics’ do not require further transfusion [1]. Iron overload from previous hyper-transfusion therapy persists unless effective iron reduction therapy (IRT) is instituted post-aHSCT. The full benefits of aHSCT in preventing long term iron related organ dysfunction can only be achieved after adequate IRT. This problem is especially more in older patients who are heavily transfused and as a result are heavily iron overloaded with organ dysfunction before aHSCT causing continued morbidity and mortality [2, 3].

It has been reported that iron overload may persist for many years after stem cell transplantation [4]. In the early days, the Pesaro Centre has used either phlebotomy or desferrioxamine (DFO) for removal of excessive body iron [2, 5] but the programme often started late, usually more than 2 years after transplantation [6]. The chronic complications of iron overload on the liver, heart and the endocrine organs are well known as well as its correlation with duration of exposure to high iron levels. Moreover, iron overload increase in early post-transplant period due to multiple blood transfusions and release of iron from marrow stores after conditioning [6]. In addition, conditioning therapy with cyclophosphamide and busulfan may increase toxic effects of iron overload on heart and liver. It is therefore imperative to institute IRT as soon as possible after successful transplantation to remove this excess iron and reduce iron related tissue damage [6].

There are only few reports with small numbers of patients in the literature on the outcome of IRT or its optimal management in ex-thalassemics. This study was thus undertaken to analyse the profile of IRT offered to ex-thalassemics transplanted at our centre and to assess their effectiveness.

### Patients and methods

Patients with TM who had aHSCT between January 2001 and December 2012, and who had IRT (phlebotomy and / or iron chelation—deferoxamine / deferasirox) with at least 3 years follow-up were included in this analysis. Patients with successful engraftment but no follow-up data available or with less than 3 year follow up and those who did not receive IRT were excluded from the analysis. Consent for analysing and publishing of data made anonymous is obtained from all patients undergoing stem cell transplant at our centre. This study protocol was approved by Institutional Review Board (IRB), Christian Medical College, Vellore, India. IRB Min No:8993 (OBSERVE] dated 04.08.2014.

Risk stratification prior to aHSCT was made as per Pesaro classification [7]. The class III patients were further stratified as Vellore low and high risk groups based on age and liver size [8]. All patients had serum ferritin levels and liver biopsy done before aHSCT. The source of graft was bone marrow for the majority of patients. Conditioning was with busulfan/cyclophosphamide or treosulfan/thiotepa/fludarabine based regimen. Graft versus host disease (GVHD) prophylaxis was intravenous cyclosporine (5mg/kg/day in 2 divided doses from day -4 with regular monitoring of drug levels) and reduced-dose short course methotrexate (10 mg/m^2^ on day +1 and 7 mg/m^2^ on days +3, +6 and +11 post-HSCT).

Patients received different modalities of IRT including phlebotomy alone, chelation alone or phlebotomy and chelation together. The chelating agents used were desferrioxamine mesylate (DFO) or deferasirox (DFX). DFO was given as subcutaneous infusion (30-50mg/kg/day for up to 5 days a week), and DFX was given at 20-40mg/kg/day orally. The major determinants for initiation of IRT were a stable graft after stoppage of immunosuppression at one year and no other ongoing long term complications such as GVHD or infections in the early years. In more recent years, IRT was also initiated earlier (as early as 5 months after aHSCT in those with adequate graft function without other complications). The type of IRT administered depended on the baseline haemoglobin (>10.5g/dl), age, ease of venous access, level of serum ferritin as well as patient / family preferences. When adequate peripheral venous access was feasible, phlebotomy, 8-10ml/Kg once in 4–8 weeks, was done only in patients with haemoglobin level was >10.5g/dl.

Clinical and laboratory assessments (including complete blood count, serum ferritin and liver enzymes) were monitored every 6–12 months during long term follow-up or as per clinician’s discretion based on patient’s clinical status. All data were recorded in prospectively maintained patient treatment and transplant records in the department of Haematology, Christian Medical College, Vellore, Tamil Nadu, India. All the data were anonymised after collecting necessary details from this primary source before statistical analysis. The details were collected during the period between January to December, 2015 and updated in July, 2018. The response to IRT was assessed based on serum ferritin levels which was monitored once in 3 months for those on regular follow-up or at the earliest opportunity after that. Reduction in serum ferritin level was calculated as a function of time. IRT was stopped at a target ferritin of <300ng/ml [9, 10] in the majority or at a ferritin level <500ng/ml in some, based on physician discretion. Patients were categorized as ‘Completed IRT’ when serum ferritin level reached the target level of <300ng/ml or <500ng/ml (in 29 patients the treating physician had stopped IRT when ferritin level was <500ng/ml but >300ng/ml due to reasons like intolerance / refusal to take chelating agent and non-feasibility of phlebotomy due to poor venous access), and as ‘Ongoing IRT’ in patients who had not reached target level of <300ng/ml and IRT was being continued at last follow up. Assessment of chimerism status was by either done by fluorescent in situ hybridisation (FISH) or variable number tandem repeat (VNTR) methods [11, 12].

Response was measured in terms of ferritin reduction per month and percentage ferritin reduction per month calculated as below;

- Ferritin reduction per month = (Ferritin at start of IRT—Ferritin at end of IRT or at last follow up for those still on IRT)/duration of IRT in months).
- Percentage ferritin reduction per month = (Ferritin at start of IRT—Ferritin at end of IRT or at last follow up for those still on IRT) ÷(Ferritin at start of IRT) x 100/duration of IRT in months).

Duration of IRT is the time in months from start of IRT to the end of IRT [for those who ‘Completed IRT]’ or till last follow up date [in those with ‘Ongoing IRT’].

#### Statistics

Descriptive statistics were calculated for all variables. Differences in proportions and two categorical variables were assessed using the chi-square test. Mann-Whitney-U test was used to compare two continuous variables. To compare more than 2 continuous variables we used Kruskal—Wallis Test. The relationships of clinical features to the outcome of the procedure were analysed by logistic regression and their 95% confidence intervals were calculated. For all tests, a 2-sided P-value of 0.05 or less was considered statistically significant. SPSS 16.0 software was used for the analysis.

## Results

A total of 281 aHSCT were performed for patients with TM from January 2001 to December 2012 of whom 68 patients either expired within day 30 of transplant or had graft failure. Of the remaining 213 patients, 149 (70.9%) fulfilled the inclusion criteria for this study and could be included in this analysis. The median follow up of this group was 80 months (range:37–182).

The baseline patient characteristics are detailed in Table 1. The median age at aHSCT was 7 years (range:1–18), and majority were males—94 (63%). Most of the patients belonged to Pesaro class III -92 (61.7%). The median number of blood transfusions prior to aHSCT was 70 (range: 10–450) and majority (65.1%) had received >50 transfusions. The median serum ferritin at the time of aHSCT was 2480 ng/ml (range: 866–8921). All except one had matched related stem cell donors, of which 143 (96%) were siblings, and majority (69.8%) had bone marrow as the source of the graft. IRT was administered as follows: phlebotomy alone—10 patients (6.7%); chelation alone—60 (40.3%) and combination—79 (53%). The median time to start IRT was 14 months (range: 5–53) from aHSCT and duration of IRT was 43 months (range: 3–136). No serious toxicities related to IRT were noted in this cohort. Liver enzymes were more than three times normal in 4 patients; 3 of whom were positive for hepatitis C virus (HCV). None of them had persistent transaminitis after completion of IRT and none had renal dysfunction during IRT. At the end of IRT, only one patient who was positive for HCV had liver enlargement >2cms below costal margin at the end of IRT.

**Table 1:** Baseline patient characteristics.

Variable	N (%)/ Median (range)
Total number of patients	149 (100)
Age (years)	7 (1–18)
**Gender**	-
Male	94 (63)
Female	55 (37)
**Lucarelli risk group**	-
Class 1	4 (2.7)
Class 2	53 (35.5)
Class 3	92 (61.7)
**No. of transfusions pre-SCT**	-
≤50	51(34.9)
>50	95 (65.1)
Donor type*	-
MRD	5 (3.4)
MSD	143 (96.0)
MUD	1 (0.7)
Stem cell source**	-
PBSC	45 (30.2)
BMSC	104 (69.8)
**Pre-IRT Ferritin (ng/ml**)	2480 (866–8921)
< 2500	75 (50.3)
>2500	74 (49.7)
**Type of IRT**	-
Phlebotomy alone	10 (6.7)
Chelation alone	60 (40.3)
Chelation + Phlebotomy	79 (53.0)
Time to start of IRT from aHSCT (mo)	14 (5–53)
Duration of IRT (mo)	43 (3–136)
**Target ferritin achieved**	-
<300ng/ml	59 (40)
<500ng/ml	88 (59)
**Complications during & post IRT**	-
Transaminitis during IRT	48 (32)
Median follow up (mo)	80 (37–182)

*MRD-Matched related donor, MSD-Matched sibling donor, MUD-matched unrelated donor.

**PBSC-Peripheral blood stem cell, BMSC-Bone marrow stem cell

### Outcome of different iron reduction therapy

**Fig 1:**
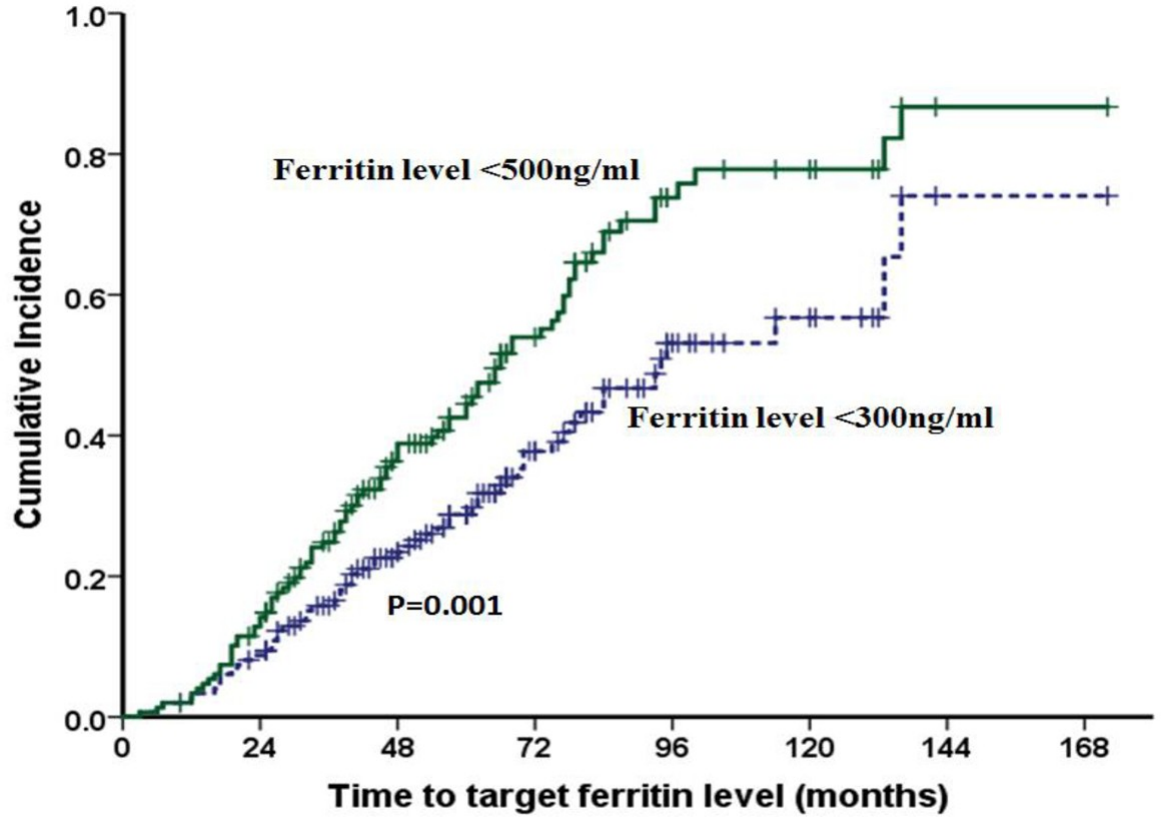
Cumulative incidence graph comparing attainment of target ferritin levels. Out of the 149 patients, serum ferritin level of <50Ong/m1 was reached in 88 (59%) patients, and target ferritin <300ng/m1 was attained by 59 (39.5%) patients in a median of 41 months (range: 3–136) of IRT.

**Fig 2:**
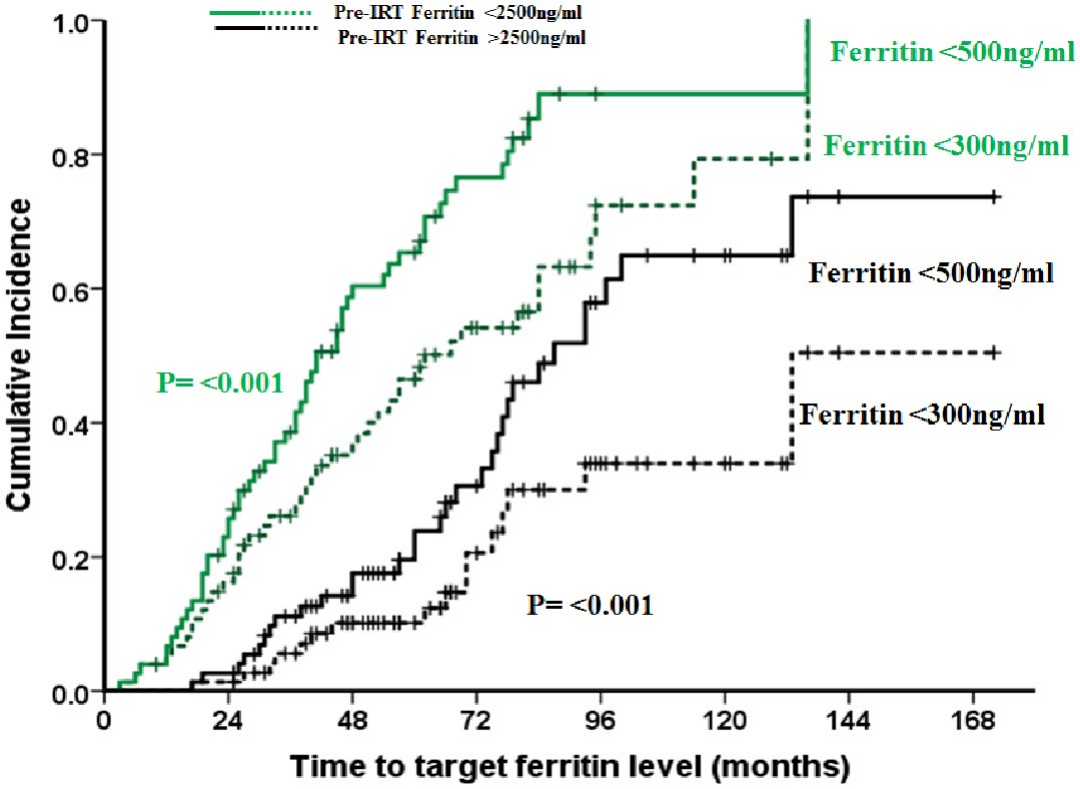
Cumulative incidence graph comparing attainment of target ferritin levels with reference to pre IRT ferritin levels. Target ferritin was achieved significantly faster when the pre-IRT ferritin level was lower (<2500ng/m1). The median time to achieve target ferritin <300ng/m1 in those with pre-IRT ferritin <2500ng/m1 (n = 43) and 2500ng/m1 (n = 16) were 39 months (range:3–136; n = 43) and 64 months (range:17–133; n = 16) respectively, whereas the median time to achieve serum ferritin level of <500ng/m1 in those with pre-IRT ferritin <2500ng/m1 and 2500ng/m1 were 37 months (range:3–136; n = 57) and 65 months (range:17–133; n = 31) respectively.

Comparison of different types of IRT and their outcomes are detailed in Table 2. Majority of patients (52.3%) were on combination of phlebotomy and chelation. The median time to start of IRT from the date of transplant were similar in all three categories of IRT; 19 months (range:5–41) for phlebotomy alone, 14 months (range:6–53) for chelation alone and 15 months (range:5–42) for combination of chelation and phlebotomy (p = 0.352). Those who received combination IRT were noted to have a higher serum ferritin at the time of stem cell transplantation (p = 0.008) compared to the other two groups, and majority (60%) in this group had ferritin >2500ng/ml. The rate of reduction of ferritin /month (ng/ml/month) was significantly higher in those who received combination therapy: 87 (range: 33–195), 130 (17–1012) and 147 (27.7–1427) in the phlebotomy alone, chelation alone and combination therapy groups respectively (p = 0.038). However, there was no significant difference in the number of patients who achieved serum ferritin level of <300ng/ml, a target recommended by the TIF [9, 10]. The chelating agents used were DFO (n = 21), DFX (n = 27) or combination of DFO+DFX (n = 13). A comparison of the these groups showed that even though more number of patients on DFX (63%) attained target ferritin of <300ng/ml compared to DFO (33.3%) and DFO+DFX (15.4%) (p = 0.010), there was no significant difference in the rate of ferritin reduction per month (S1 Table). Out of the 149 patients, at a median IRT duration of 41 months (range 3–136), 88 (59%) patients had completed IRT and were off treatment for iron over load as on last follow up. Only 59/88 (67%) patients had achieved a target ferritin of <300ng/ml, while in 29 (33%) patients IRT was stopped when serum ferritin had reached <500ng/ml. The time to achieve the target ferritin of <300ng/ml and <500ng/ml of the whole cohort, and a comparison based on the pre-IRT ferritin levels (<2500ng/ml versus ≥2500ng/ml) is shown in Figs 1 and 2 respectively. Target ferritin was achieved significantly faster when the pre-IRT ferritin was lower (<2500ng/ml). The median time to achieve target ferritin <300ng/ml in those with pre-IRT ferritin <2500ng/ml versus ≥ 2500ng/ml were 39 months (range:3–136; n = 43) versus 64 months (range:17–133; n = 16) respectively (p = <0.001, and the median time to achieve serum ferritin level of <500ng/ml in these two groups were 37 months (range:3–136; n = 57) versus 65 months (range: 17–133; n = 31) respectively (p = <0.001). On comparing those who ‘completed IRT’ versus those who were on ‘ongoing IRT’ (Table 3), patients who had completed IRT were noted be younger (p = 0.024), had lower pre-IRT ferritin levels (p = <0.001) and had lesser number of patients in Pesaro class III than those who were on ‘on going IRT’ (p = 0.043). Although on univariate analysis age, Pesaro class, number of transfusions prior to aHSCT and pre-IRT ferritin were noted to be statistically significant factors affecting response to IRT, on multivariate analysis only number of transfusions prior to aHSCT of >50 (HR = 2.3; 95% CI: (1.04–5.25); p = 0.040) and pre-IRT ferritin ≥2500ng/ml (HR = 3.3; 95% CI: (1.60–6.95); p = 0.001) retained significance (Table 4).

**Table 2:** Comparison of different types of IRT.

	Phlebotomy N (%)/ Median (range)	Chelation N (%)/ Median (range)	Chelation+Phlebotomy N (%)/ Median (range)	P value
No. of patients	10 (6.7)	61 (40.9)	78 (52.3)	-
Age	6 (3–8)	6 (1–16)	8 (1–18)	0.086
Pre-IRT ferritin	2010 (1216–2830)	2230 (1341–7660)	2775 (866–8921)	0.008
**Pre-IRT Ferritin**	-	-	-	-
<2500 ng/ml	7 (70)	37 (61)	31 (40)	0.022
≥2500 ng/ml	3 (30)	24 (39)	47 (60)	
**Lucarelli risk group**	-	-	-	-
Class 1	1 (10)	3 (5)	-	0.136
Class 2	5 (50)	19 (31)	29 (37)	
Class 3	4 (40)	39 (64)	49 (78)	
Start of IRT from aHSCT (mo)	19 (5–41)	14 (6–53)	15 (5–42)	0.352
Duration of IRT (mo)	50 (20–91)	32 (3–136)	51 (5–133)	<0.001
Ferritin reduction (ng/ml/mo)	87 (33–195)	130 (17.0–1012)	147 (27.7–1427)	0.038
% ferritin reduction/mo	4 (2.1–15.2)	5.9 (0.8–15.3)	5.5 (1.6–17.3)	0.427
**Target ferritin achieved**	-	-	-	-
<300	6 (60)	26 (43)	27 (35)	0.249
<500	8 (80)	38 (62)	42 (54)	0.228
**Time to target ferritin**	-	-	-	-
<300ng/ml	73 (26–95)	27 (3–136)	55 (12–123)	0.009
<500ng/ml	48 (33–62)	54 (23–79)	77 (39–100)	0.018
Median follow up (mo)	78 (57–135)	74 (37–151)	95 (38–182)	0.013

**Table 3:** Patient characteristics and outcome of IRT.

	IRT Status at last follow-up		P value
	Completed IRT N(%)/Median(range)	On-going IRT N(%)/Median(range)	
No. of patients	88 (59%)	61 (41%)	-
Age (yrs)	6 (1–17)	8 (1–18)	0.024
**Gender**	-	-	-
Male	53 (60%)	41 (67%)	0.490
Female	35 (40%)	20 (33%)	
**Pesaro risk group**	-	-	-
Class 1	4 (4%)	-	
Class 2	36 (41%)	17 (28%)	0.043
Class 3	48 (55%)	44 (72%)	
**No. of transfusions pre-SCT**	-	-	-
≤50	39 (45%)	12 (20%)	0.002
>50	47 (55%)	48 (80%)	
**Ferritin pre IRT (ng/ml)**	2135 (866–8921)	3130 (1500–8317)	<0.001
<2500 ng/ml	57 (65%)	18 (30%)	<0.001
≥2500 ng/ml	31 (35%)	43 (70%)	
**Time to Ferritin**	-	-	
<300ng/ml (n = 59)	41 (3–136)	-	-
<500ng/ml (n = 29)	65 (23–100)	-	
**Types of IRT**	-	-	-
Phlebotomy alone	8 (9%)	2 (3%)	0.228
Chelation alone	38 (43%)	23 (38%)	
Chelation + Phlebotomy	42 (48%)	36 (59%)	
Start of IRT from aHSCT (mo)	14 (5–42)	15 (5–53)	0.830
Duration of IRT (mo)	40 (3–136)	47 (10–114)	0.529
Ferritin reduction (ng/ml/mo)	137 (32–1012)	142 (17–1427)	0.974
% ferritin reduction/mo	6.3 (2.1–15.3)	4.2 (0.81–17.3)	<0.001
Median follow up (mo)	87 (37–166)	71 (37–182)	0.006
Ferritin at last follow up	228 (1.2–493)	1115 (518–5820)	<0.001

**Table 4:** Factors affecting response to IRT.

	Univariate			Forward Multivariate		
	HR	95% CI	P value	HR	95% CI	P value
Age (years)	1.1	1.01–1.20	0.017	-	-	-
Gender—Male	1.4	0.68–2.68	0.386	-	-	-
Pesaro risk group—Class 3	2.2	1.07–4.34	0.031	-	-	-
No. of transfusions pre-aHSCT—>50	3.3	1.55–7.11	0.002	2.3	1.04–5.25	0.040
Ferritin pre IRT (ng/ml)—≥2500	4.4	2.18–8.87	<0.001	3.3	1.60–6.95	0.001
Initial liver size—>2cms	1.8	0.81–4.02	0.149	-	-	-
Type of IRT—Chelation (ref)				-	-	-
Phlebotomy alone	0.4	0.08–2.22	0.289			
Chelation + Phlebotomy	1.5	0.77–2.80	0.318			
Start of IRT from aHSCT (months)	1.0	0.98–1.06	0.353	-	-	-
Duration of IRT (months)	1.0	0.99–1.02	0.747	-	-	-

## Discussion

The data of 149 ex-thalassemics is the largest cohort to be evaluated for IRT so far. Other large cohorts include reports on IRT by Angelucci et al (N = 48) [2], Giardini et al (N = 18) [5], and Li C et al (N = 30) [6], apart from a few other smaller series including a randomized clinical trial comparing the efficacy and safety of the oral iron chelator deferasirox versus phlebotomy over one year in ex-thalssemics by Inati et al [n = 26] and a phase II study evaluating efficacy of deferasirox by Yesilipek et al [n = 27] [13, 14]. A comparison of the present study with other major studies by Li C et al, Giadini et al and Angelucci et al is made in Table 5. The age at aHSCT is lower in our study (n = 149; age:7.4 ± 3.91 years) as compared to that reported by other authors (Angelucci et al (n = 41; 16 ± 2.9 years, p = <0.001), Li C et al; n = 30; age 10.9 ± 5.3 years, p = 0.0017) [2, 6]. In keeping with lower age group of our patients at aHSCT, the number of transfusions prior to aHSCT was lower in our study (88±66) compared to Angelucci et al (188 ±91) (p = <0.001) [2]. In spite of the younger age of the patients in this study, the risk category showed a pattern similar to other studies with the vast majority (97%) in the Pesaro class II/III risk groups in our study as well as those reported by Angelucci et al (85%), Giardini et al (100%) and Yesilipek et al (100% [2, 5, 15]. This shows the poor pre-HSCT chelation among most of the patients in India. This is further exemplified by the fact that in our cohort 62% (n = 92) were in Pesaro class III risk group and out of this 71 (77%) were in the Vellore high risk class III patients [8]. In the present study, different types of IRT alone or in combination were used, whereas phlebotomy alone was used by Angelucci et al [2], Li C et al [6] used DFO followed by either phlebotomy (14/39) or DFX (21/39), and Giardini et al [5] used DFO alone. Unal et al [16] and Yesilipek et al [14] used DFX alone.

**Table 5:** Comparison with literature.

	Present study	Li C et al (6)	Giardini et al (5)	Angelucci et al (2)
Study period	2001–2012	1992–1997	NA	1991–1992
No. of patients	149	30	18	48
Age at aHSCT (years)	7.4±3.91	10±5.3	NA	12±3.1
Pesaro class II or III	145 (97.3%)	NA	18(100%)	41(85%)
Hepatitis C virus infection	5 (3.4%)	0(0%)	14(78%)	35(85%)
Ferritin at start of IRT (ng/ml)	2480 (866–8921)	5292±3166	3607(2270–7710)	3694(2761–5246)
No. of pre aHSCT transfusions	88±66	-	-	188±91
Type of IRT used	Phlebotomy/DFO/DFX	Phlebotomy/DFO	DFO	Phlebotomy
Time to start of IRT from aHSCT(mo)	14 (5–53)	Post engraftment	NA	4.5±1.5yrs
Median duration of IRT (mo)	47±26	NA	NA	35±18
Target Ferritin for IRT (ng/ml)	300*		NA	
Patients with target (<300ng/ml) achieved	59 (40%)	NA	NA	NA
**Dose of IRT**	-	-	-	-
DFO	30-50mg/kg x 5/7	50mg/Kg	40mg/Kgx 6/7	-
DFX	20-40mg/Kg/day	-	-	-
Phlebotomy	8ml/Kg/4-6wks	10ml/Kg /4wks	-	6ml/Kg/2 wks
**Ferritin pre- IRT (ng/ml)**	-	-		
Phlebotomy group(n = 10)	1976 ± 614	2453 ± 1232	-	-
DFO group (n = 21)	*2640*±1468	5292 ± 3166		
	*P = 0*.*233*	***P = 0*.*001***		
**Ferritin at end (ng/ml)**	-	-		
Phlebotomy group(n = 10)	372±334	588 ± 234	-	-
DFO group (n = 21)	*787*±715	665 ± 733		
	*P = 0*.*043*	***P = NS***		
**Ferritin reduction/mo**	-			
Phlebotomy group(n = 10)	96±55	155 ± 82 mg/l	-	-
DFO group (n = 21)	*142*±126	405 ± 301		
	P = 0.370	***P = 0*.*001***		
**% Ferritin reduction/mo**	-	-		
Phlebotomy group(n = 10)	5.3±3.9	13±12%	-	-
DFO group (n = 21)	5.0±3.4	10±5.7%		
	P = 0.724	***P = NS***		

* In 29 patients, IRT was stopped by treating physician at a ferritin level of <500ng/ml due to one of the reasons mentioned in the methodology section. 59 patients achieved target ferritin <300ng/ml.

The median duration of IRT was longer in our cohort than that reported by Angelucci et al (47±26 months versus was 35±18 months; p = 0.006) [2]. The median duration of IRT for those who received phlebotomy alone was 50 months (range: 20–91) and for those on chelation alone was 32 months (range: 3–136) in the present study, while shorter duration was reported by Li C et al [6] (17.8 ± 8.7 months in phlebotomy group and 14±4.6 months in DFO/ DFX group). It should be noted that these comparisons are limited by the fact that the starting ferritin and the IRT protocols have been different in each of these studies. Additionally, in the present study, on comparing those who were started on IRT within 12 months versus the others (Table 6), it was noted that those who were initiated on IRT within 12 months of aHSCT were noted to have higher ferritin reduction per month and percentage ferritin reduction per month [(198.5 (18.3–1427) and 7.7 (1–17.3) versus (108 (17.02–501.7) and 4.5 (0.81–7.4) respectively; p = <0.001] compared to those initiated on IRT after 12 months post aHSCT, suggesting that early initiation of IRT helps in faster depletion of iron stores.

**Table 6:** Comparison of patients based on time of starting IRT.

	IRT within 12 months of SCT	IRT after 12 months of SCT	P value
No of patients	53 (35.6)	96 (64.4)	
Age	8 (2–17)	6 (1–18)	0.052
Pre aHSCT transfusions			
<50	18 (36.0)	33 (34.4)	
>50	32 (64.0)	63 (65.6)	0.857
Initial ferritin	2634 (1072–8244)	2351 (866–8921)	0.521
<2500 ng/ml	24 (45)	51 (53)	
≥2500 ng/ml	29 (54.7)	45 (46.9)	0.395
Ferritin reduction per month(ng/mo)	198.5 (18.3–1427)	108 (17.02–501.7)	<0.001
% ferritin reduction per month (ng/ml/mo)	7.7 (1.0–17.3)	4.5 (0.81–7.4)	<0.001
No. achieved target ferritin <300ng/ml	15 (28.3)	44 (45.8)	0.038
Time to target ferritin <300ng/ml	60 (3–172)	57 (6–136)	0.771
No. achieved target ferritin <500ng/ml	31 (58.5)	57 (59.4)	1.000
Time to target ferritin <500ng/ml	48 (3–172)	47 (6–136)	0.666

In our cohort, 59% (n = 88) had completed IRT; 40% (n = 59) achieved the target ferritin <300 ng/ml, while in 19% (n = 29) IRT was stopped at a ferritin level <500ng/ml. Reduction in serum ferritin/month ‘[absolute quantity(ng/ml/month)]’ was significantly higher in those who received combination therapy compared to phlebotomy and chelation alone (p = 0.038), while the percentage ferritin reduction per month was similar. In the observation by Li C et al [6], ferritin reduction per month was higher in chelation (DFO) group compared to phlebotomy group (p = 0.001), but the percentage reduction was not significantly higher. Our observation suggest that in order to remove iron more effectively, phlebotomy should be combined with chelation as much as possible, if feasible.

Angelucci et al [2] used annual liver biopsy along with routine biochemistry to measure iron overload post-transplant, and all patients were evaluated before and after 3±0.6 yrs of follow up; serum ferritin decreased from 2587 (2129–4817) to 417 (210–982) mcg/l (p = 0.0001) and liver iron concentration (LIC) decreased from 20.8 915.5–28.1) to 4.2 (1.6 to 14.6) mg/g dry weight (p<0.0001) [2]. In the present study and other studies mentioned above the target of completion of IRT is based on the serum ferritin levels. Serum ferritin is used as a guide to adequacy of IRT, mainly due to its easier assessment and low cost. Studies have identified that the risk of cardiac disease and death are lower when serum ferritin is maintained below 2500ng/ml [3], and an additional clinical advantage is observed when level is even lower (<1000ng/ml) [17]. Even though several studies have demonstrated that serum ferritin broadly tracks LIC [18–20], this relationship is weak in non-transfused patients [21]. It is to be acknowledged that by the time iron overload related organ failure or endocrinopathies are identified, irreversible organ damage would have set in. Hence it is important to incorporate monitoring of tissue iron overload as the final check of the adequacy of IRT in ex-thalassemics. Unfortunately, even though several non-invasive technologies are available to measure tissue iron overload, access to most of these tests are limited and they are also much more expensive [22].

The evolution of practice of IRT at our centre is summarized in Table 7. In the earlier years IRT was started in most patients at or around one year or later post-HSCT but this became much soon in more recent years. Phlebotomy was advised (8ml/kg every 4–8 weekly) in patients who had haemoglobin above 10.5g%. Various modes of IRT were used in different combinations. DFO was the only chelator used for many years in doses of 30–50 mg/kg/day 5–6 days a week. More recently, DFX is also being used in doses of 20–40 mg/kg/day. None of the patients in this cohort was advised Deferiprone (DFP) as chelating agent due its known haematopoietic toxicities. IRT is now continued till serum ferritin reaches <300ng/ml. Serum ferritin was monitored once in three to six months. No adverse events related to IRT were noted in these patients.

**Table 7:** Summary of approach to IRT at our institute.

1	When do we start IRT?	Most patients were started on IRT around 1-year post transplant (at 14 months (range: 5–53)
		Patients started on IRT within 12 months of aHSCT had significantly better Ferritin reduction per month and % Ferritin reduction per month than those started on IRT after 12 months
2	How do we do IRT?	a) Phlebotomy -8-10ml/Kg Q 4–6 weeks
		b)DFO– 30-50mg/kg/day x 5/7 days; s/c
		c) DFX– 20-40mg/kg /day
		d) DFO/DFX + Phlebotomy
		e) DFO+DFX ± Phlebotomy
3	In which patients do we start phlebotomy?	Phlebotomy is done in patients with Hb maintained >10.5g%
4	How frequently do we advise phlebotomy?	In majority, once in 4–6 weeks (Not documented in all patients)
5	How long do we continue IRT?	Till the serum ferritin reaches <300ng/ml
6	How frequently do we monitor serum ferritin while on IRT?	Not uniform In majority, it is done once in 3–6 months depending on their follow up frequency
7	Which is the better IRT observed in our cohort?	Probably Chelation + Phlebotomy (those on Phlebotomy+Chelation seems to have the maximum Ferritin reduction/month

Based on this study and the other data available in the literature, it may be concluded that IRT needs greater attention after aHSCT and should be initiated at the earliest in ex-thalassemics with a combination of methods to reduce ferritin to target levels. The following may be considered for IRT in ex-thalassemics:

1. IRT may be considered early in the post aHSCT period (6 months or earlier) if patient has a stable graft and no significant GVHD.
2. Combination of phlebotomy + chelation is likely to reduce iron faster particularly in those with high serum ferritin at the start of IRT if they maintain a haemoglobin of >10.5g% post-aHSCT.
3. Phlebotomy may be considered safe if a steady haemoglobin is maintained of >10.5g%.
4. The aim of IRT should be to bring serum ferritin to the normal range at the earliest possible time.
5. Serum ferritin may be monitored every 3–6 months during IRT and IRT regimen may be intensified as needed.
6. Stopping of IRT should be based on achieving the target serum ferritin level in the normal range and combined ideally with confirmation of no iron overload in critical organs.

However the many questions pertaining to IRT in ex-thalassemics still remain unanswered. There are no guidelines on initiation and intensity of IRT in ex-thalassemics or its stoppage with long term follow-up data. Large prospective studies comparing various IRT modalities and serial monitoring of iron load are required. Furthermore, follow up studies on these patients who have completed IRT, to assess the clinico-pathological effects of tissue iron overload on the overall growth and organ functions, can help determine the optimum time for IRT initiation and completion as well as the ideal modalities to assess successful completion.

## Supporting information

S1 Table Comparison of the different chelators. (TIF)

S1 Data Minimal data sheet. (XLSX)
