# Supplementary figures and images for: Outcome of iron reduction therapy in ex-thalassemics
Source: PLoS One. 2021 Jan 22;16(1):e0238793. doi: 10.1371/journal.pone.0238793 (PMC7822270; doi:10.1371/journal.pone.0238793)

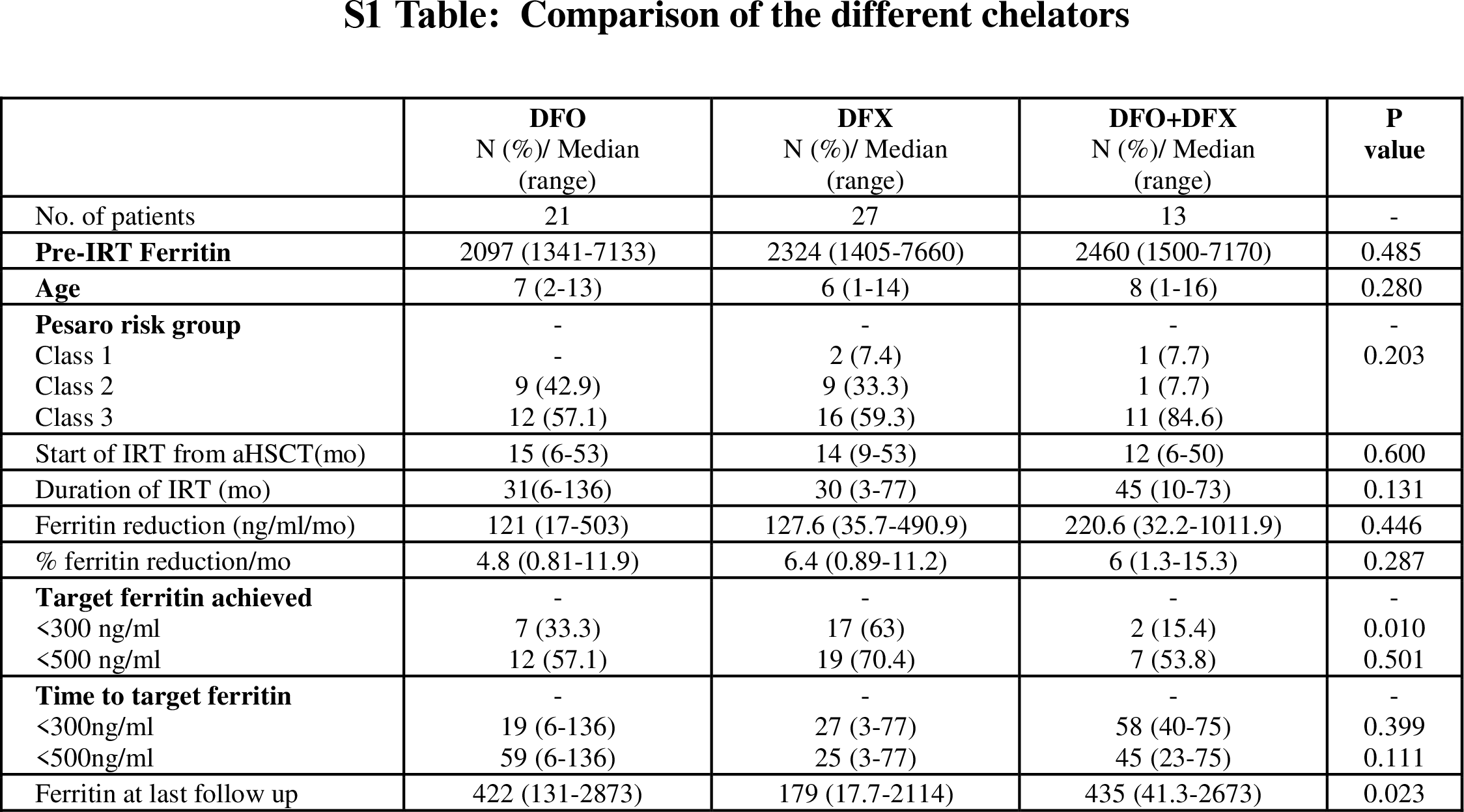

Supplement: S1 Table — (TIF) [file pone.0238793.s001.tif]
